# Person-Centered Care in Glioblastoma: The Art of Early Advance Care Planning

**DOI:** 10.3390/cancers18030413

**Published:** 2026-01-28

**Authors:** Jennifer Serventi, Nimish Mohile

**Affiliations:** Department of Neurology, University of Rochester Medical Center, 601 Elmwood Avenue, Rochester, NY 14642, USA; nimish_mohile@urmc.rochester.edu

**Keywords:** advance care planning, glioblastoma, end-of-life, cognition, decision-making, quality-of-life 6, patient-centered

## Abstract

Advance care planning (ACP) is fundamentally important for patients diagnosed with glioblastoma (GBM), a highly aggressive primary brain tumor with a grim prognosis. The urgency for early ACP is profoundly amplified by the characteristic, progressive neurocognitive decline that frequently impairs critical reasoning and leads to the loss of decisional capacity. ACP is a proactive process ensuring that future medical interventions align with a patient’s deeply held values and goals. Proactive ACP discussions are associated with less aggressive end-of-life (EOL) care, improved quality of life for patients and care partners, earlier hospice enrollment, and reduced psychological distress for surrogate decision makers. Despite guidelines recommending early integration, ACP prevalence remains low due to clinician discomfort with EOL discussions, a perceived lack of adequate training, and a widespread “culture of shared avoidance”. Experts recommend initiating ACP at or shortly after diagnosis, normalizing it as standard cancer care. Using structured communication strategies, such as the REMAP tool, and empowering allied health providers to champion these conversations are key integration strategies. Ultimately, early and skillful ACP is an ethical imperative that safeguards patient autonomy and minimizes the burden on loved ones.

## Simple Summary

Advance care planning is a crucial component of patient-centered care for patients with serious illnesses. This is critical for patients with glioblastoma due to the terminal nature of the disease, as well as the potential for rapid decline and early cognitive dysfunction, which can negatively impact decision-making capacity. Benefits and barriers to advance care planning conversations are well documented, but there is little prospective research in patients with glioblastoma. Providers who care for patients with glioblastoma should understand the importance of advance care planning, develop comfort with these conversations and be aware of various roadmaps and tools to enhance the process.

## 1. Introduction

Advance care planning (ACP) is a fundamental process that empowers individuals and their care partners to thoughtfully consider, discuss, and formally document their preferences for future medical care and life-sustaining interventions. This proactive approach ensures that healthcare interventions closely align with a patient’s deeply held values, goals, and preferences. Ideally, these conversations occur earlier in care, especially for patients who receive terminal diagnoses.

Historically, many clinicians have hesitated to initiate ACP discussions due to concerns that such conversations might cause psychological distress, diminish hope, or increase anxiety. However, evidence has shown that engaging in ACP does not decrease hope or increase hopelessness and may even reduce anxiety among patients with advanced cancer [1]. Given the complex clinical picture of glioblastoma (GBM), characterized by significant morbidity, as well as its high symptom burden and inevitable impact on cognition, healthcare must proactively balance life-prolonging therapies with preserving quality of life and facilitating timely, appropriate ACP [2]. Experts advocate for standardizing ACP as an integral part of comprehensive cancer care and recommend initiating these discussions at or shortly after diagnosis. American Society for Clinical Oncology (ASCO) guidelines for patients with advanced cancer recommend that patients be referred for palliative care services that focus on understanding and education about illness and prognosis, clarification of treatment goals, and assistance with medical decision making—which are all components of advance care planning [3]. The European Association for Neuro-Oncology (EANO) has published guidelines calling for advance care planning and the use of advanced directives early in the disease trajectory for patients with malignant glioma [4]. ACP can provide patients with a forum to explore their values and goals in the context of a serious diagnosis, modeling true patient-centered care.

To establish a robust clinical agenda, this review is grounded in Kitwood’s Person-Centered Care Theory. This framework emphasizes that for individuals with progressive neurocognitive decline, timely interventions are essential to maintain personhood and dignity. In the context of GBM, early ACP serves as a mechanism to preserve the patient’s voice before decisional capacity is compromised.

## 2. Methodology

This manuscript is a narrative expert review. The authors performed a literature search using databases including PubMed and Google Scholar, focusing on peer-reviewed articles published between 2000 and 2025 related to glioblastoma, advance care planning, and palliative care. Selection was based on clinical relevance to neuro-oncology and the strength of evidence regarding end-of-life (EOL) outcomes.

## 3. The Clinical Context of Neuro-Oncology

Glioblastoma is the most common high-grade primary central nervous system tumor in adults, affecting 7.14 people per 100,000 annually in the U.S. [5]. Patients typically present with a wide array of neurological symptoms, including headaches, personality changes, hemiparesis, seizures, and significant cognitive and communication impairments. Cognitive symptoms can rapidly worsen in the first few weeks to months after diagnosis. Patients who do not initially present with cognitive symptoms frequently go on to develop them as a consequence of treatment, medications and tumor recurrence. Despite standard treatment protocols involving maximal safe surgical resection, external beam radiotherapy, and chemotherapy with temozolomide, survival remains dishearteningly short with a median overall survival ranging from 12 to 21 months and a strikingly low 10-year survival rate of 0.71% [6,7,8].

As previously mentioned, a defining characteristic of GBM is the early and progressive cognitive decline experienced by patients. Studies tracking neurocognitive functioning in patients with high-grade glioma (HGG) reveal a marked deterioration over time, particularly between 8 and 16 months from diagnosis [9]. This decline affects critical cognitive domains such as information processing, psychomotor function, attention, verbal memory, working memory, and executive functioning—all of which profoundly impact decision-making capacity. In turn, it is challenging for patients to comprehend complex medical information, deliberate about treatment options, and understand the impact of their choices. Furthermore, clinicians often overestimate a patient’s true cognitive capacity [10], which underscores the need for structured capacity assessments prior to discussions about treatment options and goals of care. Because clinicians often overestimate a patient’s cognitive capacity, we recommend incorporating routine cognitive screening tools such as the Montreal Cognitive Assessment (MoCA) or Mini-Mental State Exam (MMSE) into clinical practice. The inherent risk of losing decisional capacity underscores the importance of early ACP to ensure that patients’ wishes are captured while they are still able to articulate them, thus helping to alleviate some of the decisional burden for health care proxies and family members.

Cognitive decline in malignant gliomas is often multifactorial, including both the impact of treatments such as surgery and radiation, but also the intrinsic nature of the tumor itself, disrupting normal neuronal networks that are essential for cognition [11]. Beyond the direct impact of the tumor and interventional treatments, medications such as antiepileptic drugs [9] and chemotherapy also contribute to cognitive changes for patients with HGG [12].

Living with GBM also carries a heavy emotional and psychological toll for both patients and their families [13,14]. Patients frequently report worries about the future and the distressing loss of everyday skills [15]. Family care partners (CPs) often experience psychological distress comparable to, or even greater than, that of the patient themselves and those caring for patients with advanced cancer provide about 8 h of daily assistance with symptom management, emotional support, personal care, and complex care coordination, which leads to immense emotional strain, compromised physical health, and even increased mortality risk [16]. Care partners commonly report that they feel unprepared for their daily tasks and struggle with significant financial difficulties [15,17]. Given the overall cognitive and emotional impact of a glioblastoma diagnosis for patients and their families, early ACP provides an opportunity to relieve some of that burden. 

## 4. Rationale and Benefits for Early Advance Care Planning

The compelling rationale for early ACP in GBM is rooted in its proven ability to enhance patient-centered care and mitigate adverse outcomes for both patients and their families. Proactive ACP discussions are demonstrably associated with less aggressive and invasive care at the end of life, resulting in improved quality of life for both patients and care partners, earlier hospice enrollment, reduced psychological morbidity, and care that is truly aligned with patient preferences [18]. While the timing of ACP is often debated, we provide a practical working definition of “early ACP” as discussions initiated at or shortly after diagnosis, ideally within the first two months and prior to the first disease recurrence. Conversely, when individuals defer ACP and subsequently lose decision-making capacity, they often undergo unwanted medical treatments, incur unnecessary costs, endure needless suffering, and inadvertently place an overwhelming burden on their loved ones [1].

Despite the known risk of cognitive decline, a substantial number of patients with GBM—as many as 27%—lack documentation of any advance directives before their last month of life, and nearly a quarter do not have detailed treatment-limiting orders until their final week [2]. In addition, because many advance directives are completed late in the disease course, studies have shown that almost half of Medical Orders for Life Sustaining Treatment (MOLST) forms are consented for by surrogate decision makers and not by the patients themselves [2]. This places an onerous burden upon families and care partners to make decisions for their loved one, often without a true understanding of their loved one’s preferences. It is well known that surrogate decision makers experience significant guilt, emotional distress, and decisional conflict surrounding this role. Not surprisingly, data shows that having advance directives in place for patients who are critically ill directly reduces surrogate decision makers’ stress and indirectly lessens depressive symptoms [19]. 

Based on this evidence, both the ASCO and the EANO guidelines have recommended that ACP and the use of advance directives should be incorporated early in the trajectory of serious oncologic diagnoses, including GBM; however, the definition of “early” remains up for debate [4,20]. The benefits of early ACP in cancer and specifically in patients with GBM cannot be overstated. First, early ACP safeguards patient autonomy by ensuring their healthcare wishes are articulated, documented, and respected, even when they are no longer able to speak for themselves [2,21]. As a patient’s cancer progresses and appropriate treatment options are exhausted, it is common for patients and their families to search desperately to find anything that could buy more time. It is in this period of desperation that some patients pursue more aggressive care without any clear evidence of benefit. ACP is associated with fewer hospital admissions at the end of life [22], which, in turn, reduces unnecessary healthcare costs [1]. Not surprisingly, one study of patients in a high-risk primary care program linked proactive ACP discussions with lower total medical expenses in the last six months of life, equaling more than $2500 per month, as well as a three-fold increase in hospice enrollment [23].

As previously mentioned, advance care planning specifically tailored for care partners has demonstrated significant benefits, including lower depression scores and reduced stress burden, particularly in the crucial period leading up to the patient’s death [16]. Such interventions also contribute to better care partner preparedness and may alleviate financial strains by avoiding unwanted aggressive medical interventions and visits [15,17].

The ethical and legal right of competent adults to make decisions regarding life-sustaining medical treatment is well-established in the U.S. [1]. Advance care planning serves as a cornerstone for achieving goal-concordant care, upholding the principles of informed consent and substituted judgment, reducing significant psychological distress for families, and ultimately ensuring care aligned with the patient’s best interests [15].

## 5. Current Gaps and Barriers

Despite the compelling rationale, significant gaps and persistent barriers continue to hinder comprehensive ACP implementation for patients with GBM. The documented prevalence of ACP among GBM patient cohorts remains alarmingly low and highly variable, ranging from 4% to 55% across different studies and countries [7,24]. In a notable study, only 29% of patients with GBM had ACP documented within six months of diagnosis, with the rate increasing to 55% only by the time of death [7,25].

### 5.1. Clinical Gaps and Barriers

A primary obstacle is clinicians’ widespread discomfort with EOL discussions, which they often perceive as emotive, time-intensive, and requiring an elusive “right window of opportunity” as well as special skills or training [1,12]. This discomfort leads to avoidance, as clinicians fear causing patients pain, anxiety, or inadvertently taking away hope [1]. In contrast, much of the available data tells a different story. Results of a randomized controlled trial of a systematic ACP tool indicated a 50% reduction in anxiety and depression in oncology patients [26]. Patients reported a better understanding of their prognosis, closeness with their clinician and improved ability to focus on planning for the future. 

Like oncology providers, family members often share the same concerns and discomfort around ACP. The hesitation from both parties can result in confusion over who should initiate these visits—will the clinician indicate when the appropriate time arrives and offer the conversation or is the onus on the patient and family to request an ACP conversation? This deferral can then result in missing a window when the patient can still actively contribute. 

Additional provider-related barriers include the challenge of providing accurate survival predictions for patients with advanced cancer, sometimes adopting an overly optimistic stance that can inadvertently lead to unrealistic expectations of care goals and hastier decision-making at the EOL due to deferred ACP [15]. Moreover, many healthcare professionals acknowledge a lack of confidence and adequate training in conducting sensitive, emotive ACP discussions. Some feel “out of their depth” when addressing these complex conversations, and there are few opportunities for practice and skill-building outside of actual clinical care [12].

### 5.2. Systemic and Cultural Barriers

While ACP is widely recognized as a shared responsibility among multidisciplinary teams, this concept is often misconstrued in practice as someone else’s responsibility, resulting in a pervasive “culture of shared avoidance” [12]. This diffusion of responsibility leads to a lack of accountability and collective inaction on the part of the medical team. This is an inherent risk in the care of patients with GBM who optimally are being cared for by a multidisciplinary team that may include a (neuro)oncologist, neurosurgeon, radiation oncologist, primary care provider, palliative care specialist and multiple advanced practice providers. With a team of this size, one could easily assume another provider will address the “elephant in the room” and take on the task of discussing advance directives and EOL decisions.

Furthermore, in busy clinical environments where time is a scarce resource, the time-intensive nature of ACP discussions often relegates them to a lower priority, resulting in missed opportunities or hurried conversations [12]. Additionally, there is an ambiguity in the definition of ACP. Many healthcare professionals, particularly nurses and allied health professionals, express uncertainty about what specific practices constitute ACP and how it differs from their existing roles in discharge planning or general patient support. Surgeons and other physicians often see their role solely as one of treatment and may designate ACP as the role of allied health professionals, given the relationships they often form with patients and families and their ability to focus on the softer aspects of care. This ambiguity contributes to the lack of a clear framework for implementation in clinical practice [12].

There is a perception among some clinicians that initiating EOL discussions could disrupt patients’ and families’ optimistic outlook or imply that the provider is not being fully transparent about the diagnosis and prognosis. Finding the ideal time to introduce ACP discussions is challenging since we want to ensure that patients and families have sufficient understanding of the diagnosis, but have not yet lost decision-making capacity or experienced an unplanned medical crisis that necessitates these decisions to come into play. Given the rapid and often unpredictable nature of brain tumors, these ideal moments can be rare and fleeting.

## 6. Clinical Strategies for Integration of Early ACP

To overcome these barriers and foster patient-centered care, a systematic and proactive approach to ACP is essential, particularly for patients with GBM. ACP discussions should ideally be initiated at or shortly after diagnosis for patients with newly diagnosed high-grade primary brain tumors. It may take several rapport-building encounters for patients and families to accept the benefit of such a conversation; therefore, normalizing ACP early can be beneficial. Key triggers for initiating or revisiting ACP include the delivery of a terminal diagnosis, disease progression, signs of cognitive impairment or language dysfunction, and a change in a patient’s functional status. If a patient displays cognitive deficits early in the disease course, there should be an urgency for the ACP visit. Given the unpredictable and rapid nature of cognitive decline in GBM, regularly revisiting ACP discussions is crucial as clinical events unfold or the patient’s status changes. 

A collaborative approach involving the entire multidisciplinary team is paramount to avoid the culture of shared avoidance. Adopting a model in which providers from a specific specialty claim responsibility for ACP discussions can formalize the process, allowing them to increase their comfort and confidence in moderating ACP conversations and creating champions of ACP within the medical team. Advanced practice providers, social workers and clinical nursing staff are poised to be excellent champions of ACP discussions. These professionals have in-depth clinical knowledge, are adept at patient education, often spend more time than their physician counterparts in communication with patients and families, thereby solidifying rapport, and may have more flexibility in their clinic schedules to allow sufficient time for ACP discussions. Empowering these non-physician professionals to engage and lead these discussions can benefit patients and the medical system alike. 

### 6.1. Structured Communication Strategies

When setting up ACP discussions, providers should be thoughtful in their approach, never assuming the patient and family understand the purpose of ACP or the provider’s motivation for encouraging the visit. It can be helpful to frame the discussion as an opportunity for the provider to discuss the big picture and for the patient to consider their values and priorities in the context of their diagnosis, given that much of our time in medical visits is dedicated to addressing symptoms, reviewing test results and making treatment decisions. Normalizing ACP by reminding patients that even healthy adults should have these conversations with their loved ones can be helpful as well. Patients should be encouraged to invite close family and friends to be part of this important conversation, including family care partners and particularly their surrogate decision makers. 

**Figure 1:**
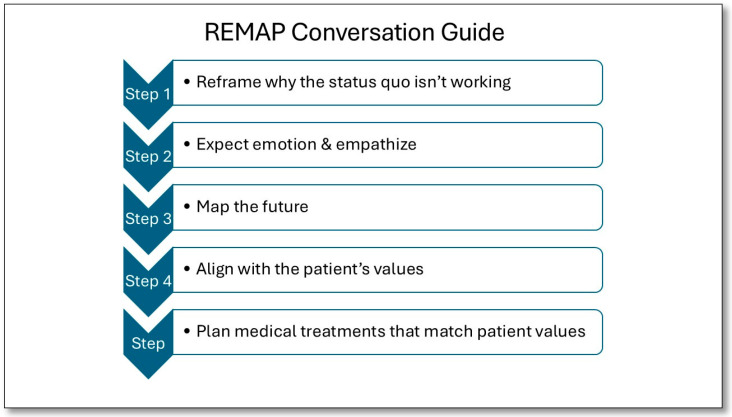
The REMAP conversation structure.

**Figure 2:**
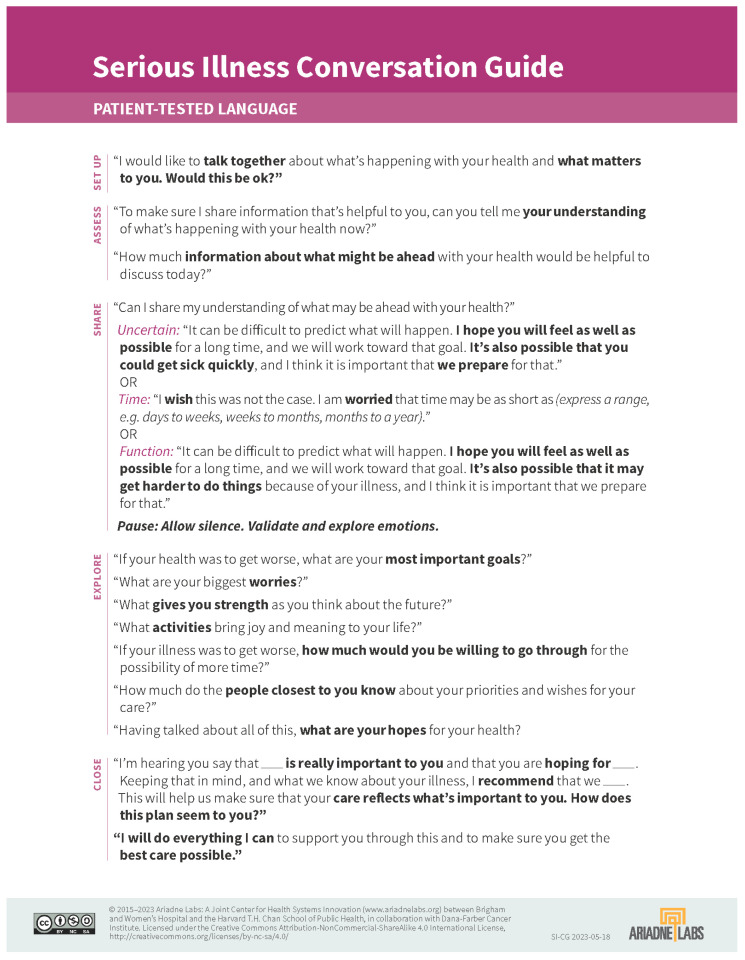
The Serious Illness Conversation Guide.

**Figure 3:**
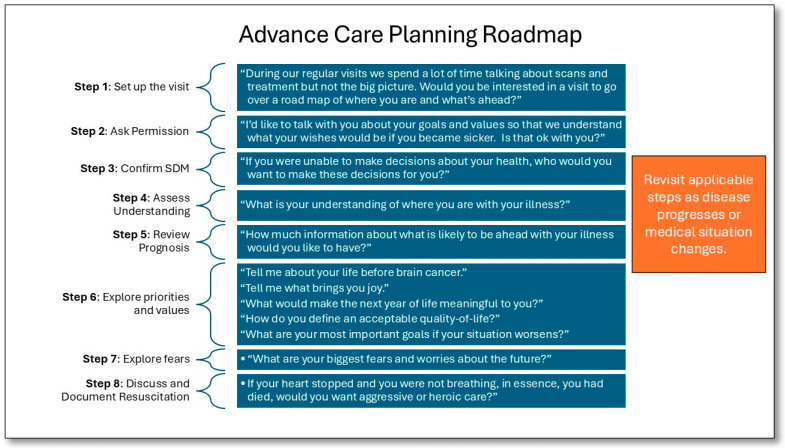
Advance care planning roadmap steps and suggested open-ended questions to facilitate discussion.

We recommend using a structured, phased approach, such as the REMAP tool (Figure 1), which can guide providers through difficult conversations by using steps to Reframe, Expect Emotion, Map Outpatient Values, Align with Values, and Propose a Plan [27]. Other available frameworks include the Serious Illness Conversation Guide [28] (Figure 2) and the SPIKES Protocol for Breaking Bad News [29]. Our Neuro-oncology group at the James P. Wilmot Cancer Institute uses a similar ACP checklist and guide (Figure 3). This includes asking permission to discuss difficult topics, confirming the role and identity of surrogate decision makers, assessing the patient’s understanding of their diagnosis, reviewing prognosis and realistic expectations, defining the patient’s priorities, values and fears, addressing cardiac and respiratory resuscitation options and expected outcomes, and finally allowing time to discuss options for end-of-life settings with associative expectations of care partners. When patients are amenable, our guide also allows us an opportunity to describe a typical death from brain cancer, which, in our experience, has allayed fears and provided patients and their families with a sense of understanding and preparedness. These tools or roadmaps guide individual patient ACP conversations while also providing a framework for clinicians to become more adept at moderating these complex conversations. Additionally, it is helpful to be prepared with open-ended questions that can help facilitate each step of the conversation (Figure 3).

#### The Advance Care Planning Road Map

We begin the visits by negotiating the agenda to set expectations and establish a conducive environment. Some families like the physical closeness of an exam room, while others may prefer a virtual visit setting so that they can be at home in familiar and comforting surroundings. We routinely instruct patients and their families that we have no specific goal or expected outcome for the visit outside of providing them with accurate information about their disease and reasonable expectations of what their outcomes may be in specific medical situations. As providers, we serve as guides to assist them in navigating their choices and documenting any decisions they make.

The next step is to assess the patient and/or family’s readiness to discuss goals of care, which helps understand willingness to engage in EOL discussions. It is critical to ask for permission to discuss difficult topics. This provides the patient with a sense of control over the conversation and the pace. Existing advance directives (e.g., Power of Attorney, Health Care Proxy or Surrogate Decision Maker, Medical Orders for Life Sustaining Treatment (MOLST) forms or Physician Orders for Life Sustaining Treatment (POLST) forms), which vary by state, should be reviewed in detail to ensure they accurately reflect current wishes. While ACP is a global ethical imperative, specific tools like POLST/MOLST are US-centric. An essential step in ACP visits is to establish the patient’s preferred substitute decision maker and ensure that they and the patient understand the appropriate applications of and limitations of the role. 

We explore the patient’s understanding of their medical situation and recognize that it is common to assume the patient understands more about their diagnosis than they actually do. To accomplish this, we ask open-ended questions to the patient, such as, “tell me your understanding of your diagnosis and where you are in your illness”. Patients with GBM should be able to identify that their cancer is not curable and terminal. If the patient’s understanding is accurate, confirmation is provided, but if not, we seek permission to discuss prognosis and clearly explain how long they may be expected to live and what that life is expected to look like. Clarifying the specific role and goal of treatments (e.g., curative, palliative, time-buying at a cost) can also be helpful. This level of detail provides a realistic understanding crucial for informed decision-making [1].

Once all attendees have a level understanding regarding the severity of the diagnosis and expected outcomes of medical care, there is an opportunity to explore the patient’s values, goals, priorities, hopes, fears, and concerns for the future. This is essential to person-centered care. We ask leading questions about what their life was like prior to their cancer diagnosis and what activities they enjoyed. We solicit from the patient and their family any important or pressing “bucket list” goals which may include a family trip they have always wanted to take, completing a project they have started, repairing relationships, or simply spending as much quality time with family and friends as possible. We can then explain which goals are realistic and which are not in the context of the patient’s specific disease and functioning and encourage families to move forward with plans for trips and events. Attempts to offer clarity on the timing of such events, allowing families to make or change plans as needed (e.g., moving up a wedding date so the patient can attend), can be particularly meaningful.

Another meaningful role of ACP discussions is to clarify how individual patients define “quality of life”. What symptoms, side effects or deficits are they willing to tolerate if they can still get more time? Are there disabilities that are unacceptable to them, and where is their so-called “line in the sand” for continuing cancer-directed treatments? By providing specific scenarios, patients can more tangibly understand what life might be like with various deficits and symptoms, allowing them to envision a resultant quality of life. We find it helpful during this stage for families to hear directly from the patient what they are willing to tolerate. We can offer scenarios such as the patient being wheelchair- or bed-bound, being unable to effectively communicate, needing full care, or residing in a skilled nursing facility. Having a clear understanding of this can aid providers during treatment decision discussions to ensure that options are encouraged that align with the patient’s QOL preferences. This may be most helpful for family members who are struggling with a patient’s decision to withhold certain aspects of care or move towards comfort measures only care plans.

Soliciting the patient’s and/or family’s fears can be informative, as we may alleviate those fears through our experience with prior patients and knowledge of the disease process. Common fears include that of painful languishing at the end-of-life and concerns over being a burden to family CGs. Describing a typical death from GBM educates patients about common EOL symptoms and how we manage them and provides space for family care partners to respond to patient concerns surrounding the burden of their care. We may take some time to explain the role that Hospice plays at the end of life and clarify that Hospice is an approach to care rather than a place. Hospice services can be provided in an array of settings and reviewing these options with the patient and family can clarify the patient’s preferences for where they wish to die (e.g., home, nursing facility, comfort care home, or in the hospital).

Finally, when addressing cardiac and respiratory resuscitation options, it is essential to explain the actual steps taken during these interventions, offer scenarios that could potentially lead to cardiac arrest and/or respiratory distress, and explore realistic survival and functional outcomes. The public perception of the success of emergency resuscitation is largely inaccurate, with people overestimating survival rates, likely due to imprecise portrayals in media such as television and movies [30]. Over 50% of laypersons estimate cardiopulmonary resuscitation (CPR) to be successful for cardiac arrest in upwards of 75% of cases, while the American Red Cross reports the CPR success rate is 10% for out-of-hospital incidences and 21% for in-hospital events [31]. Data shows that patients who possess an accurate understanding of successful cardiac resuscitation rates are more likely to refuse resuscitation interventions [32]. Additionally, we should forecast what deficits or limitations the patient might endure should they survive a cardiac arrest. It is reasonable to specifically estimate the likelihood that the patient would be weaned from a ventilator, leave the hospital, require a skilled nursing facility placement, or regain independence with meaningful quality of life. 

We take a similar approach in discussions of respiratory arrest and distress, describing the common conditions in patients with GBM that may necessitate intubation and ventilation, including but not limited to severe infections, pulmonary embolism, and status epilepticus. Similarly, providing our best estimate for reasonable recovery from these ailments can assist patients in imagining themselves in those scenarios and, therefore, help inform their decisions about life-sustaining treatments. At the end, we summarize our understanding of the patient’s wishes and help them document their wishes in the appropriate forms, which can vary by country and state. 

ACP is not a one-time encounter but occurs on a continuum [33]. A patient’s values and priorities may change over time as milestones are reached, disease progresses, or the goals of available treatments change. Revisiting ACP periodically throughout a patient’s health trajectory ensures their currently documented wishes remain accurate. Patients should be informed and reminded that these decisions are not final and that they can and should be readdressed at future visits and with changes in clinical functioning and disease state.

## 7. Role of Palliative Care in Facilitating ACP

Palliative care (PC) plays a crucial and distinct role in facilitating comprehensive ACP for patients with any serious illness, including GBM. It is important to distinguish between primary palliative care, which is the basic symptom management and ACP provided by the neuro-oncology team, and specialist palliative care, which involves a dedicated team for complex physical, emotional, or existential distress. It is also vital to differentiate palliative care, which focuses on symptom management and maximizing quality of life for patients with serious illness, with hospice care, which is typically reserved for patients in the last 6 months of their lives who wish to forgo life-extending treatments and transition their focus to comfort measures only. Clarifying that these two services can be delivered in tandem or separately may help patients be more accepting of PC services. 

Early integration of PC has been consistently linked to improved quality of life and significant reductions in physical and psychological symptom burden for patients with cancer. ASCO guidelines explicitly recommend that patients with advanced cancer receive dedicated palliative care early in their disease course [34]. Despite these recommendations, actual rates of PC involvement in GBM management remain low (39–40%) [15,25] and most consults occur during inpatient hospital admissions for acute issues [2]. Reasons for this hesitancy to involve PC include concerns about burdening the patient with additional clinical teams and visits, worry that patients may develop feelings of rejection at being “out-sourced” [35], and a desire on the part of the primary oncologist or neuro-oncologist to manage these conversations themselves [36]. Neuro-oncology providers are uniquely positioned to lead these discussions due to their deep understanding of the disease trajectory and their longitudinal relationships with patients and families. It is the responsibility of the clinical team to guide families through these challenging social and medical situations.

Neuro-oncology providers should consider how PC referrals can supplement the care they already provide. Despite not having neuro-oncology-specific training, PC providers are experts in emotional and social support, management of symptoms such as depression and fatigue, and are skilled at navigating complex family dynamics. Additionally, face-to-face time is often limited for neuro-oncology providers, and though primary ownership of palliative and supportive care lies with them, PC providers may provide additional complementary opportunities to address other existential issues with grace and precision.

## 8. Research Directions and Future Perspectives

The existing body of literature on ACP in GBM reveals critical gaps and inconsistencies, underscoring the urgent need for robust prospective research. There is a wide array of available retrospective data, but existing prospective evidence is sparse, with a notable lack of randomized controlled trials specific to advance care planning for patients with glioblastoma. Future studies should aim to address questions regarding the overall timing of ACP in patients with GBM, evaluate patient and family experiences in ACP, assess the efficacy of specific existing interventions, and address provider and patient barriers to these visits occurring in a timely manner. Given the unique neurocognitive challenges experienced by this patient population, there is also a need to develop and validate disease-specific ACP tools that consider common communication deficits and could easily be incorporated into ACP discussions for patients with GBM. There may be opportunities to leverage digital and AI-based tools to serve as valuable resources supporting patients and families in navigating complex treatment and end-of-life decisions. 

Training programs for medical teams, including advance practice providers, social workers and nursing staff, should be widespread, accessible, and strongly encouraged to improve comfort and build skill in ACP discussions. Education should also include appropriate documentation of advance directives and the details of the patient’s expressed wishes. Centers should consider providing education surrounding billing for these visits. Although these visits take time, many states will provide reimbursement on time for advance care planning discussions. 

## 9. Recommendations and Expert Opinion

As providers in the neuro-oncology space, we guide our patients and their families through the diagnosis of terminal and life-altering illnesses, difficult prognoses, complicated treatment decisions and challenging social situations. There is a pressing need for patients with serious illnesses, like GBM, to be offered and provided with skilled discussions surrounding these decisions. 

We therefore recommend the following key points:

- Providers should advocate for ACP initiation shortly after diagnosis, preferably prior to disease recurrence. This proactive approach is essential in this patient population, who are at particularly elevated risk of rapid cognitive decline and who may lose the ability to actively participate in ACP discussions.
- Providers should normalize ACP as part of comprehensive, standard cancer care. This will help destigmatize the process and foster a philosophy where planning for the future is expected and supported by all parties.
- Institutions should consider standardizing approaches to ACP for all patients with serious illness, implementing clear protocols and offering provider education to address discomfort and prevent the culture of shared avoidance.
- Physicians should recognize that allied health providers are well-poised to moderate ACP visits with GBM patients and should support advanced practice providers, social workers and nursing staff as champions of these discussions. This can increase access to ACP for patients and families and potentially elevate job satisfaction within the team.
- Providers should remember that ACP is not an isolated conversation or visit. ACP occurs continuously, with values and priorities potentially changing with time and changes in health.

## 10. Implementation Framework

In order to facilitate proactive and skilled ACP discussions with patients with GBM and other serious illnesses, we suggest the following implementation framework and implementation pathway (Table 1):

Key Initiators: Identifying ACP Champions

To avoid a “culture of shared avoidance” where each team member assumes another will lead the discussion, roles must be clearly defined.

- Primary Clinicians: Neuro-oncologists, medical oncologists, and radiation oncologists are uniquely positioned to lead these discussions due to their longitudinal relationships with the patient. Multidisciplinary teams should be encouraged to choose who will take the lead.
- Advanced Practice Providers (APPs), social workers, and clinical nursing staff are highly effective leaders for these visits. They often have more flexible schedules and a strong rapport with families. Consider them the primary champions.
- Specialist Referrals: Palliative care (PC) should be involved early, especially for complex physical, emotional, or existential distress.

Timing and Clinical Triggers

Introduce ACP at or shortly after diagnosis, ideally within the first two months and prior to the first disease recurrence.

Triggers to initiate or revisit ACP include:

- Diagnostic: Delivery of a terminal or life-altering diagnosis.
- Clinical: Disease progression or a significant change in functional status.
- Cognitive: Signs of cognitive impairment, language dysfunction, or poor results on routine screening tools like the MoCA or MMSE.
- Personal: The patient reaching a significant life milestone or a change in treatment goals.

The Step-by-Step Implementation Pathway

Use a structured framework like REMAP, the Serious Illness Conversation Guide, or a framework similar to the following:

## 11. Conclusions

A glioblastoma diagnosis is devastating for both the patient and their family. From the beginning, patients struggle with a lack of control in the wake of challenging physical and cognitive symptoms, intense treatment regimens and a large amount of uncertainty. Engaging these patients in skillfully delivered advance care planning is not merely a recommendation but an ethical imperative. Early and proactive ACP serves as a valuable tool to define goals and values, preventing unwanted suffering and providing patients with some control over their future. Similarly, these conversations are often a gift to families and surrogate decision makers, potentially preventing them from having to make “best guesses” in times of medical crises. They no longer must see themselves as solely a decision-maker but instead can assume the role of their loved one’s “voice”, simply carrying out decisions the patient had previously made surrounding their own care. 

By systematically incorporating ACP discussions in routine care, becoming adept at moderating them through education and practice, and normalizing ACP for patients and families, we can create a culture that prioritizes patient autonomy, dignity, quality-of-life and peace of mind. When these conversations are had skillfully, we demonstrate empathy, kindness, compassion, and consideration for who our patients are as human beings. 

## Figures and Tables

**Table 1:** The Step-by-step Implementation Pathway.

Step	Action and Recommended Language
Set the agenda	Negotiate the visit’s purpose; frame it as the “big picture discussion” rather than reviewing treatments and test results
Ask permission	Ask for permission to discuss difficult topics to give the patient a sense of control
Confirm Proxies	Identify proxies or surrogate decision makers; ensure they understand their role
Assess understanding	Use open-ended questions like, “What is your understanding of your diagnosis and where you are at with your illness?”
Review prognosis	Clarify if disease is curable vs. treatable and what the goals of treatment are (cure, palliative, buying more time)
Map values	Explore the patient’s “line in the sand”—the deficits or side effects that they are unwilling to tolerate
Address Fears	Discuss fears of patient and family; explain how symptoms will be managed
Resuscitation	Provide realistic data on CPR/intubation success rates and expected clinical outcome of survival to inform decision making
Document	Assist patient in documenting decisions with applicable forms
Revisit	ACP is not a one-time event but is a continuum of care. Readdress with changes in health, quality-of-life, disease status or once life goals/milestones have been reached

## Data Availability

No new data were created or analyzed in this study. Data sharing is not applicable to this article.
